# Predicting Forefoot-Orthosis Interactions in Rheumatoid Arthritis Using Computational Modelling

**DOI:** 10.3389/fbioe.2021.803725

**Published:** 2021-12-23

**Authors:** Emily S. Kelly, Peter R. Worsley, Catherine J. Bowen, Lindsey S. Cherry, Bethany E. Keenan, Christopher J. Edwards, Neil O’Brien, Leonard King, Alex S. Dickinson

**Affiliations:** ^1^ School of Engineering, Faculty of Engineering and Physical Sciences, University of Southampton, Southampton, United Kingdom; ^2^ School of Health Sciences, Faculty of Environmental and Life Sciences, University of Southampton, Southampton, United Kingdom; ^3^ Cardiff School of Engineering and Cardiff University Brain Research Imaging Centre, Cardiff University, Cardiff, United Kingdom; ^4^ University Hospital Southampton NHS Foundation Trust, Southampton, United Kingdom

**Keywords:** foot orthosis, computational modeling, FEA, tissue strain, deep tissue injury, foot

## Abstract

Foot orthoses are prescribed to reduce forefoot plantar pressures and pain in people with rheumatoid arthritis. Computational modelling can assess how the orthoses affect internal tissue stresses, but previous studies have focused on a single healthy individual. This study aimed to ascertain whether simplified forefoot models would produce differing biomechanical predictions at the orthotic interface between people with rheumatoid arthritis of varying severity, and in comparison to a healthy control. The forefoot models were developed from magnetic resonance data of 13 participants with rheumatoid arthritis and one healthy individual. Measurements of bony morphology and soft tissue thickness were taken to assess deformity. These were compared to model predictions (99th% shear strain and plantar pressure, max. pressure gradient, volume of soft tissue over 10% shear strain), alongside clinical data including body mass index and Leeds Foot Impact Scale–Impairment/Footwear score (LFIS-IF). The predicted pressure and shear strain for the healthy participant fell at the lower end of the rheumatoid models’ range. Medial first metatarsal head curvature moderately correlated to all model predicted outcomes (0.529 < *r* < 0.574, 0.040 < *p* < 0.063). BMI strongly correlated to all model predictions except pressure gradients (0.600 < *r* < 0.652, *p* < 0.05). There were no apparent relationships between model predictions and instances of bursae, erosion and synovial hypertrophy or LFIS-IF score. The forefoot models produced differing biomechanical predictions between a healthy individual and participants with rheumatoid arthritis, and between individuals with rheumatoid arthritis. Models capable of predicting subject specific biomechanical orthotic interactions could be used in the future to inform more personalised devices to protect skin and soft tissue health. While the model results did not clearly correlate with all clinical measures, there was a wide range in model predictions and morphological measures across the participants. Thus, the need for assessment of foot orthoses across a population, rather than for one individual, is clear.

## Introduction

Rheumatoid arthritis (RA) is frequently characterized by deformities at the metatarsophalangeal (MTP) joints, frequently with erosion and subluxation of the first and fifth MTP joints (Bowen et al., 2011). Where RA has resulted in hallux valgus deformities, lateral displacement of the sesamoid bones is common (Nix et al., 2012). The surrounding soft tissues are also affected, with the plantar fat pad migrating distally from under the metatarsal heads increasing their vulnerability (Jaakkola and Mann, 2004). Bursae also pose problems, through inflammation or adventitial bursae formation as a response to friction and high pressures (Bowen et al., 2010; Van Hul et al., 2011). Associated with these morphological changes are pain, reduced foot function, and risk of soft tissue wounds (van der Leeden et al., 2006; Bowen et al., 2011). The most common sites of ulceration in the RA foot are the dorsal aspect of hammer toes (48%), the metatarsal heads (32%), and the medial aspect of the first MTP joint, with many ulcers reoccurring (Firth et al., 2008).

Generally, people with RA experience increased forefoot peak pressures compared to healthy individuals during weightbearing (Rosenbaum et al., 2006; Bowen et al., 2011; Carroll et al., 2015). Increased pressure has been shown to correlate to joint erosion and damage (Tuna et al., 2005; van der Leeden et al., 2006), but not pain, swelling or disability measures (van der Leeden et al., 2006; Konings-Pijnappels et al., 2019). Additionally, there may be limited associations between external pressures, which are easily measured, and internal tissue stresses, which are more indicative of injury (Linder-Ganz et al., 2007). Using Finite Element (FE) Analysis in other scenarios, elevated internal soft tissue stresses have been predicted around bony prominences with low soft tissue coverage, where compression also generates shear stresses (Takahashi et al., 2010). Many studies comparing plantar pressures to RA-related factors, such as pain or disability, are limited by measuring pressures barefoot rather than shod. Shoe choice is important in treating RA, as poorly-fitting footwear cause high pressures at the bony prominences of the metatarsal heads, particularly where joints are deformed (Woodburn and Helliwell, 1996).

Foot orthoses (FOs) are also prescribed to improve quality of life (QoL) by offloading painful regions. However, literature on the effectiveness of FOs in RA treatment is unclear. The effects of several footwear and orthosis variables upon plantar/forefoot pressure and pressure-time integrals (PTIs) have been studied experimentally, including shoe choice (Hennessy et al., 2007), FO customization (Hennessy et al., 2012), orthosis stiffness/rigidity and adaptations including metatarsal bars and domes (Tenten-Diepenmaat et al., 2019), and optimizing FOs based on pressure measurements (Tenten-Diepenmaat et al., 2020). These interventions typically reduced pressure but did not have a clear effect on pain or disability scores. One study found that FOs only significantly reduced peak pressures in a sub-group who had high in-shoe pressures without an FO (Tenten-Diepenmaat et al., 2020). Thus far, no single FO design is considered best practice to reduce pain and improve QoL in RA treatment, and the focus has primarily been on plantar pressure reductions even though other factors are involved (Hooper et al., 2012).

Computational modelling provides additional understanding of how loading affects the soft tissue and joints internally. Modelling studies have assessed the effectiveness of FO designs, though the studies tend to assess healthy individuals rather than those who would use the FOs (Chen et al., 2003; Cheung and Zhang, 2005; Goske et al., 2006). These models vary in configuration, using 2D or 3D geometry, linear elastic or hyperelastic properties for the soft tissue and orthosis, and a single bulk soft tissue group or including ligaments and tendons (Cheung et al., 2009). Another consideration is inclusion of the shoe. Despite evidence that shoe uppers also affect soft tissue loading (Woodburn and Helliwell, 1996; Dahmen et al., 2020), most studies only include the sole (Chen et al., 2003; Spirka et al., 2014; Chen et al., 2015; Telfer et al., 2017). Other studies have evaluated FO behavior without including the shoe (Cheung and Zhang, 2005; Zhang et al., 2020). As most of these models were based on a single healthy individual, the results relate only to that individual and may not apply to symptomatic populations, such as people with RA. There have been some exceptions where studies have assessed a pathological individual or multiple people. One study modelled a person with midfoot arthritis (Zhang et al., 2020), and another optimized FO design for 18 people with diabetes (Telfer et al., 2017). However, no studies have assessed people with RA, whose pathology, and thus orthotic requirements, differ from those with diabetes or post-traumatic midfoot arthritis.

In the present study, models were developed for people with RA affecting the forefoot. The aim was to ascertain whether simplified forefoot models would produce differing biomechanical predictions depending on RA severity, and relevant anatomical measures from magnetic resonance (MR) imaging. The RA model predictions were also compared to model predictions from a healthy individual for contrast. Such models would provide advantages over experimental studies where it is difficult to examine internal effects of loading, and pave the way for FO and footwear choices tailored to the requirements of individuals with RA.

## Materials and Methods

This study involved secondary analysis of patient imaging and clinical outcome data sets from established studies (FeeTURA) (Cherry et al., 2014) by image-based computational modelling.

### Participants

MR and questionnaire data were obtained, which had been collected from participants with RA during FeeTURA (Cherry et al., 2014), including assessments of condition severity through subjective means such as the Leeds Foot Impact Scale. The scale consists of two subsections: the impairment/footwear section (LFIS-IF) includes pain and footwear choices, while the second section covers activity limitation and participation restriction (LFIS-AP) (Helliwell et al., 2005). Instances of bursae between and beneath the MTP joints were also recorded, along with joint erosion and synovial hypertrophy. Detailed methodology for how these parameters were determined was published previously by Cherry et al. (2014). Of the original 30 participants for whom MR data were available, 13 (aged 29-73, all female) were selected for the present study, purposively sampled to represent a range of LFIS-IF scores. These participants will henceforth be referred to as P1-13. The purpose of this study was to assess the performance of the models across a range of condition presentations. As such, the selection of participants was not controlled in any other way, so that potentially influencing factors would not be excluded.

A healthy individual, with no known medical conditions or history of musculoskeletal injuries to the foot and ankle, was assessed for comparison (female, aged 31 years). MR data for this individual had been collected in a previous study at the Cardiff University Brain Research Imaging Centre (CUBRIC, Dr Bethany Keenan). Ethical approval was granted by the relevant institutional and local authority committees for the original studies and secondary analysis purposes.

The participant selection and subsequent segmentation, measurement and FE modelling processes were completed by different researchers (ASD and ESK respectively) so that the study could be carried out blinded to the participants’ reported condition severities.

### MR Segmentation and Morphological Measurements

MR data for the RA participants had been collected specifically for the FeeTURA study in 2010–2011. The MR sequencing is detailed in the FeeTURA study in full (Cherry et al., 2014). The T1-weighted spin echo sequence taken in the coronal plane was used for this study (repetition time/echo time (TR/TE): 656 ms/15 ms, slice thickness: 3mm, in-plane resolution: 0.52 × 0.52 mm). The distal slices consisting of only the toes were removed, leaving only the metatarsal and MTP joint regions. MR data for the healthy volunteer had been collected with a Dual Echo Steady State (DESS) sequence in the sagittal plane (TR/TE: 13.48 ms/4.79 ms, resolution: 0.6 × 0.6 × 0.6 mm). This was carried out using a 3T scanner (Magnetom Prisma, Siemens, Erlangen, Germany) with a foot/ankle and four-channel flex coil with the plantar forefoot rested against a flat support.

**FIGURE 1:**
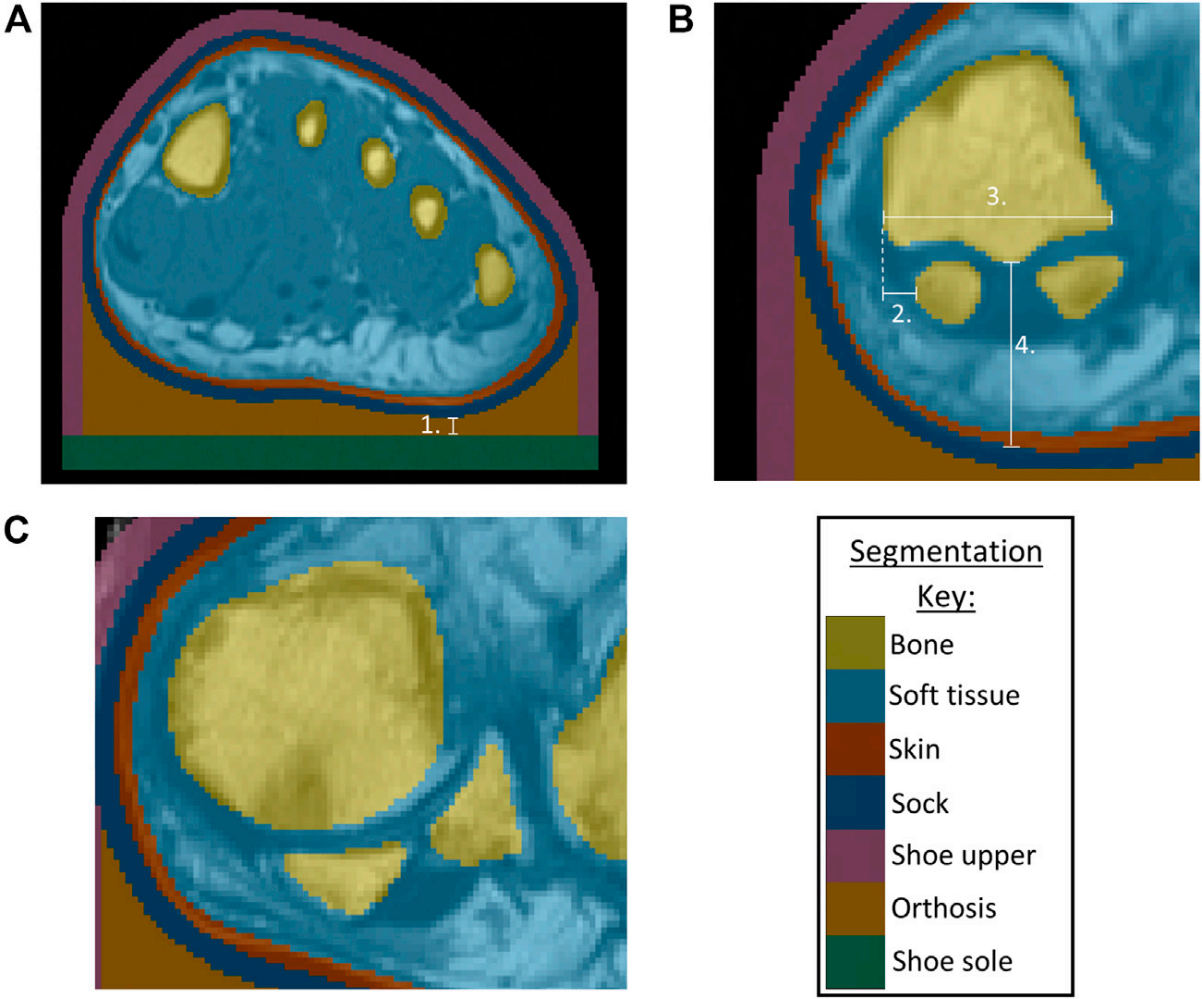
**(A)** Segmentation of bones, skin, soft tissue from P3 MRI, with addition of sock, shoe upper, orthosis, shoe sole, indicating minimum orthosis depth (1.) **(B)** Morphological measurements for P3 showing sesamoids in a normal position, with 2. Lateral distance from MH1 edge to sesamoid, 3. MH1 width, 4. Tissue depth under MH1 **(C)** P1 MRI showing example of sesamoids in a displaced position.

The MR data were segmented using ScanIP (Simpleware ScanIP N-2018.03-SP2 Build 55, Synopsys, Mountain View, United States). The RA participants’ MR data had been collected using a standardized foot position, with the coronal slices perpendicular to the metatarsal parabola. However, as the data were not originally collected for modelling purposes, the images were not orientated within the coronal plane, in positions appropriate for gait. Thus, the data were rotated in the coronal plane using ScanIP to ensure all the forefoot sections were appropriately and similarly orientated, using the metatarsal heads as references. Masks were created for the skin, underlying soft tissue and bone using greyscale thresholds. The same threshold values were used across all participants except for the healthy volunteer, where the values were adapted to suit the different MR sequencing that had been used. Where the scans included the proximal phalanges, these were fused to the metatarsals. A 2 mm thick sock was generated by dilating the skin mask in-plane. This was repeated to form a footwear model, representing a simplified leather shoe. This was given a 3 mm thick upper and 6 mm sole, allowing space for a total contact FO with a 3 mm minimum depth (Figure 1A). The simplified foot anatomy present in these models was deemed appropriate as the study purpose was comparison between models built using the same methods, rather than determination of absolute values.

Morphological measurements were taken using ScanIP’s linear measurement tool, to be used as potential indicators of condition severity (Figure 1B). The first metatarsal head (MH1) region was investigated as a key bony prominence, potentially increasing the risk of damage to the surrounding tissue. Assessments were made regarding the sesamoid bone orientations, observing whether they were neutrally positioned under MH1 (normal position) or shifted laterally beyond MH1 (displaced position) (Figure 1C), and measuring the lateral distance from the medial edge of MH1 to the medial edge of the sesamoid. This measure was presented as a percentage of MH1 width. The depth of tissue under MH1 was measured to indicate tissue migration. Surface meshes of MH1 were exported to MATLAB (R2020b, MathWorks, Massachusetts, USA), to calculate the average principal curvature of the medial plantar quarter of MH1, using code sourced from MathWorks File Exchange (Rusinkiewicz, 2004; Ben Shabat and Fischer, 2015).

### FE Model

**FIGURE 2:**
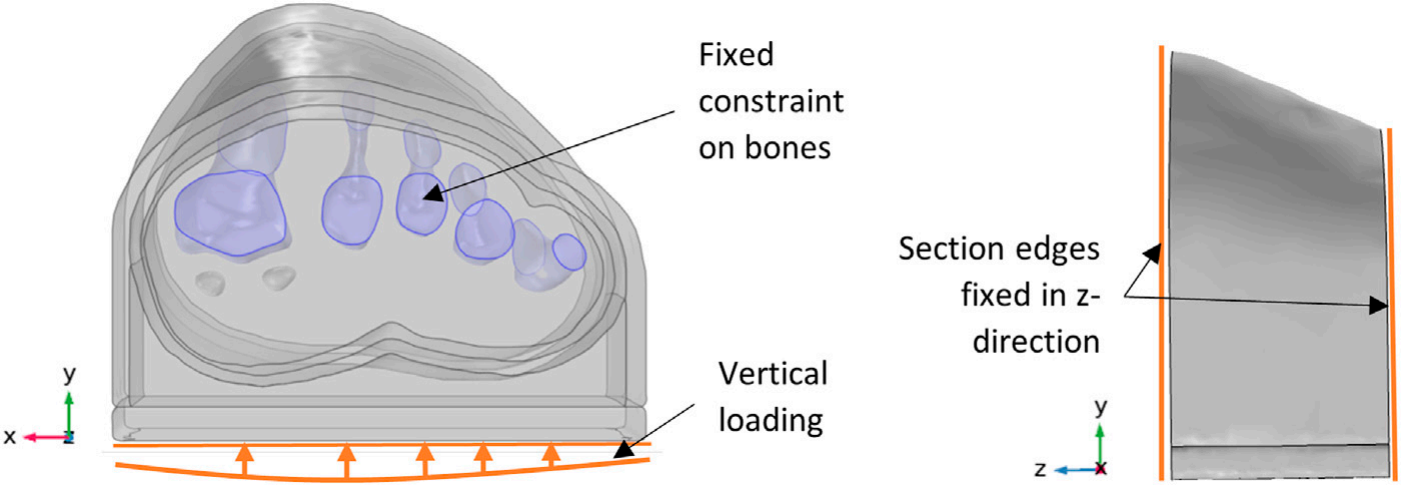
P3 model, indicating vertical loading and boundary conditions.

The segmented geometries were imported into COMSOL Multiphysics v.5.5 (COMSOL Inc., Stockholm, Sweden) for FE analysis (Figure 2). Material properties of the shoe upper, sock and bone were assumed to be linear elastic (Table 1). The skin and soft tissue were modelled using a first order Ogden hyperelastic model for incompressible materials, using the following strain energy function $W_{s}$ :

$$W_{s}=\;\frac{\text{µ}}{\alpha }\left(\lambda_{1}^{\alpha }+\lambda_{2}^{\alpha }+\lambda_{3}^{\alpha }-3\right)$$ (1)

The orthosis and shoe sole materials were modelled using Storakers model for highly compressible foam:

$$W_{s}=\;\frac{2\text{µ}}{\alpha^{2}}\left(\lambda_{1}^{\alpha }+\lambda_{2}^{\alpha }+\lambda_{3}^{\alpha }-3+\frac{1}{\beta }\left(J_{e\tau }^{-\alpha \beta }-3\right)\right)$$ (2)

For both equations, µ represents the shear modulus, α the deviatoric exponent, β the volumetric exponent, $J_{el}$ the elastic volume ratio, and $\lambda_{i}$ the principal stretches in each direction.

**TABLE 1:** Material Properties for linear elastic shoe sides, sock, bone and hyperelastic soft tissue, orthosis, shoe sole.

Material	Elastic parameters		Hyperelastic parameters			References
	Young’s modulus (MPa)	Poisson’s ratio	µ (kPa)	α	β	
Shoe upper (leather)	200	0.3	—	—	—	Goske et al. (2006)
Sock (cotton)	1.8	0.4	—	—	—	Zhou et al. (2010); Tian et al. (2019)
Bone	7,300	0.3	—	—	—	Cheung and Zhang, (2005)
Skin	—	—	452	5.6	—	Ahanchian et al. (2017)
Soft Tissue (exc. skin)	—	—	36	4.5	—	Ahanchian et al. (2017)
Orthosis (Poron)	—	—	144	4.013	0.057	Petre et al. (2006)
Shoe sole	—	—	1,588	7.708	0.292	Petre et al. (2006)

A fixed constraint was applied to all bones except the sesamoids, which were allowed to move freely as they would anatomically where they are embedded within the flexor hallucis brevis. The cut boundaries of the modelled foot section were assigned a fixed displacement perpendicular to the cutting plane. Friction was applied between the orthosis and upper and the sock with a static coefficient of 0.55 (Carlson, 2006). The orthosis, shoe sole and upper were bonded together, as were the bones, soft tissue and skin.

Vertical and medial ground reaction forces (GRF) were applied to the external boundary of the shoe sole in contact with the ground, to represent midstance of gait. The medial force was 5% of the participant’s body weight (Jung et al., 2016). The vertical force was scaled, using the participant weight and comparative length of forefoot section:

$$GRF_{vertical}\left(N\right)=Participant\;Weight\left(kg\right)\times 9.81\;\times \frac{Forefoot\;section\;length}{Full\;foot\;length}\times 1.4$$ (3)

The multiplier of 1.4 represented the proportion of load distributed to the forefoot during midstance (van der Leeden et al., 2006). This single load value was adjusted across the forefoot’s width, using multipliers of 1.05, 1.26, 1.06, 0.88 and 0.75 for the load under the first to fifth metatarsal heads, respectively (Figure 2). This distribution was determined using gait pressure data of healthy individuals, collected with F-scan in-shoe sensors (Tekscan, Massachusetts, USA), and agreed with literature for people with RA (van der Leeden et al., 2006).

A second order tetrahedral mesh was used with local refinement in the narrow skin and sock domains, and at the orthosis/sock boundary (Appendix 1). Mesh convergence was assessed using P1 models. For the mesh used, percentile shear strain and pressure results were within 2.5% of the finest mesh results. Similarly, a sensitivity analysis was performed to ensure that the proximity of the forefoot section’s cut edges to the region of interest would not affect the model results (Appendix 2).

### Data Analysis

Four parameters were used to provide indications of tissue damage risk, that had been used previously in literature (Oomens et al., 2013; Bader and Worsley, 2018; Steer et al., 2021). Data from the models were processed in MATLAB to calculate the following:

1. 99th percentile shear strain (calculated from Green-Lagrange strain tensor) in the soft tissues,
2. volume of tissue above 10% shear strain,
3. 99th percentile plantar pressure,
4. maximum plantar pressure gradient.

The percentile calculations were based on soft tissue volume for shear strain and orthosis/limb interface area for pressure. 99th% values were used rather than maximums so that outliers due to highly localized peaks were excluded and so did not affect the model comparisons. The percentile value itself (99th) was chosen based on examination of the relevant histograms, so that the majority of the pressure or strain was included. For the pressure gradient calculations, the plantar pressure data was resampled to a 5 × 5mm resolution to reflect experimental F-scan sensor measurements. The pressure results along the cut edges of the forefoot sections were omitted from this resampling process as they were affected by the boundary conditions. The maximum pressure gradient was then found by calculating the pressure difference between each point and its neighbours, divided by the distance between points. Alongside these model results, clinical data for the participants were assessed including body mass index (BMI), LFIS-IF scores, instances of bursae, erosion and synovial hypertrophy at the joints, and the MRI-based anatomical measures mentioned above. For some analyses, the BMI was grouped into normal BMI (18.5 ≤ BMI <25 kg/m^2^) and high BMI (BMI ≥25 kg/m^2^).

SPSS Statistics (v.27, IBM Corp., Armonk, NY, United States) was used to carry out statistical analyses of the model results and participant data. Shapiro-Wilk normality tests were performed to determine whether parametric or non-parametric tests were appropriate. Disease duration, lateral sesamoid offset and volume of tissue over 10% shear strain were found to be non-parametric. The remaining FE model predictions, morphological measurements and clinical data were found to be parametric. Pearson correlation analyses were performed to determine the relationships between parametrically distributed variables, and where variables were skewed, Spearman’s correlations were used.

## Results

### Demographic and Morphological Comparisons

**FIGURE 3:**
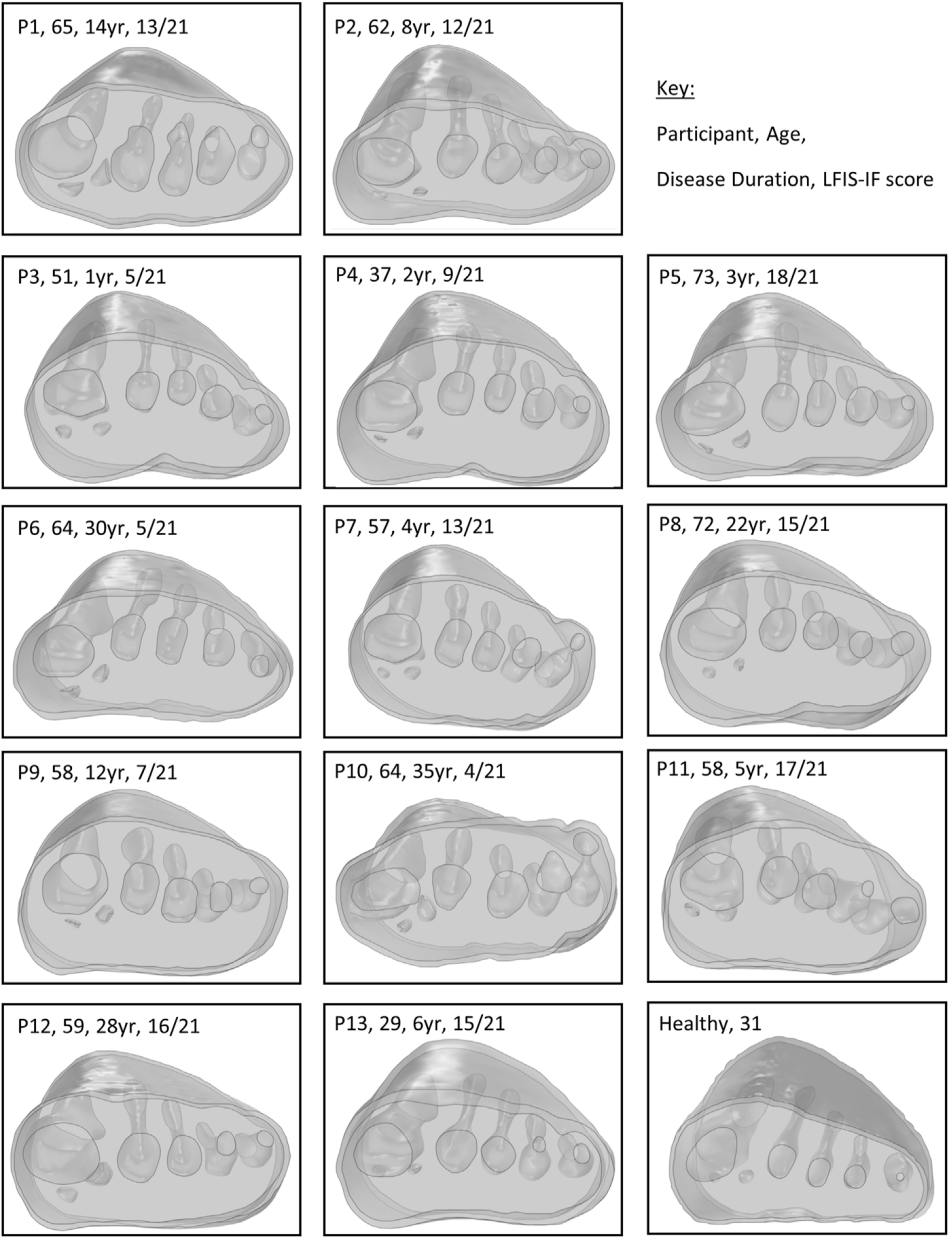
Modelled forefeet (skin, encapsulated bulk soft tissue, bones) of the 13 participants with RA and one healthy participant, with age and disease duration indicated.

**FIGURE 4:**
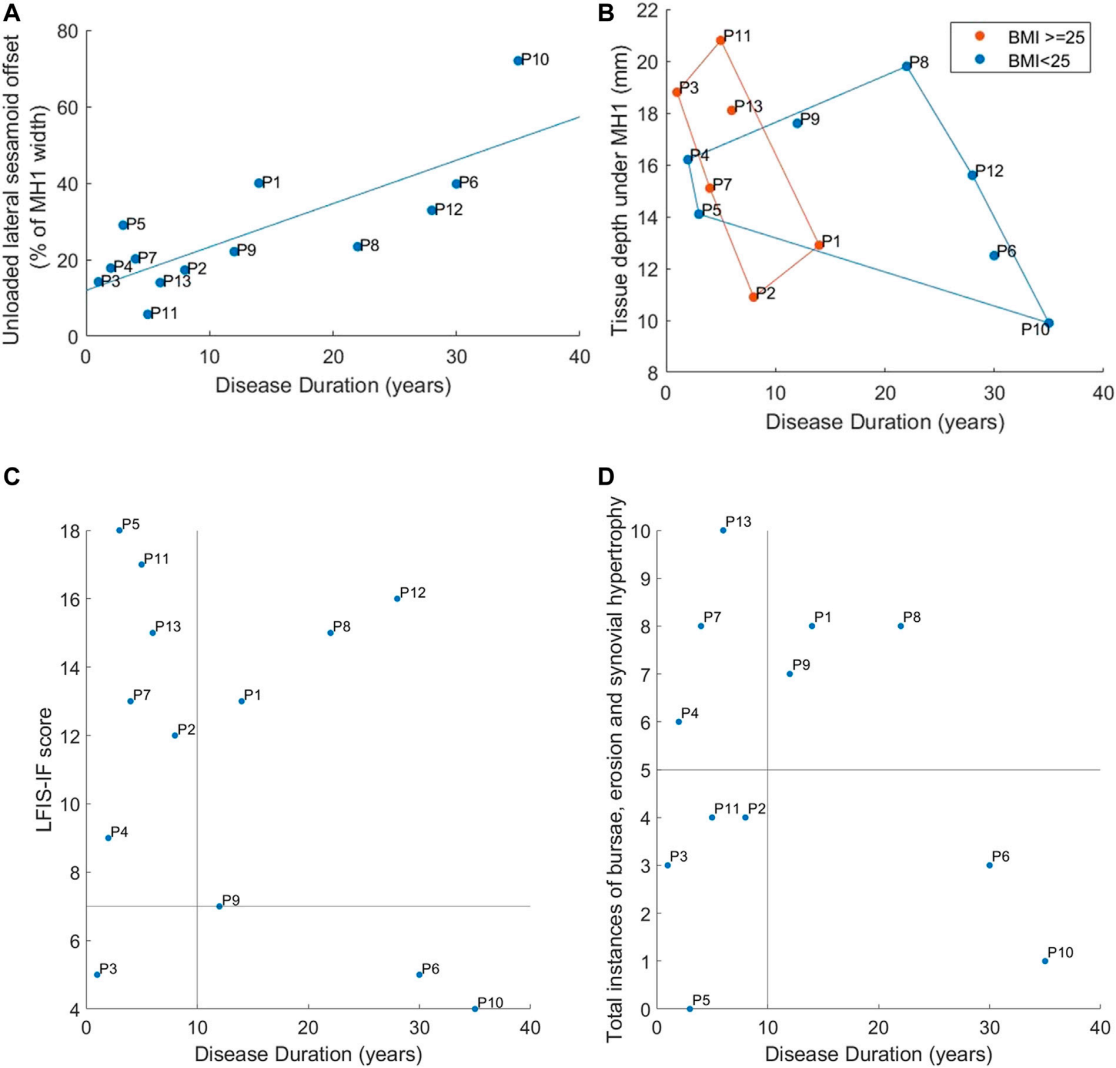
Correspondence of disease duration to: **(A)** Lateral offset of the sesamoid bones **(B)** Tissue depth under MH1 with groupings for normal vs high BMI using marker colors and convex hull boundaries **(C)** LFIS-IF score with quadrants indicating short vs long duration and low vs moderate/high LFIS score **(D)** Instances of bursae, erosion and synovial hypertrophy at the MTP joints, with quadrants showing short vs long duration and low vs high instances. Note: data for P12 was missing for instances of bursae, erosion and hypertrophy and so n = 12.

All 13 RA participants selected for this secondary analysis had relatively established disease, of duration over 1year and varying morphological presentations (Figure 3). Longer disease duration was correlated with sesamoid bones offset (*r* = 0.698, *p* = 0.008) (Figure 4A). The sesamoid offset also correlated with soft tissue depth under MH1 (*r* = -0.721, *p* = 0.005). Longer disease duration did not correlate significantly with reduced soft tissue depth, potentially due to the confounding effects of foot size and BMI (Figure 4B). Despite this, longer disease duration was not necessarily associated with a worse clinical presentation (Figure 4C, D). For example, in participants with RA for over 10 years, there was an even split of people with low vs moderate to high foot impairment (LFIS-IF threshold of 7 (Turner et al., 2006)). P6 and P10 had low LFIS-IF scores and instances of bursae, erosion and hypertrophy despite their longer disease durations and thus higher sesamoid offset and lower tissue depth under MH1. The opposite was observed for P1 and P8.

### RA and Healthy Model Comparisons

The healthy participant had a normal BMI and sesamoid bones in a neutral position, confirming their suitability as a control. The healthy participant’s FE model predictions generally fell at the lower end of the RA models’ range, either just within or below the inter-quartile range (IQR) (Table 2). The exception was the pressure gradient predictions where the healthy participant fell towards the upper end of the IQR. Note that n = 12 for instances of bursae, erosion, and synovial hypertrophy, as data was unavailable for P12.

**TABLE 2:** Median (IQR) results for the clinical data, morphological measurements and model predictions. The healthy participant rank indicates where they fell within the RA dataset, with one being lowest and 14 highest. X = denotes where rankings where tied.

	Measure	RA participants (n = 13)	Healthy participant (n = 1)	
		Median (IQR)	Median (IQR)	Rank (out of 14)
Clinical Data	BMI	24.8 (22.3–28.4)	22.3	4 =
	Disease duration (years)	8 (4–22)	NA	NA
	LFIS-IF (0-21)	13 (7–15)	NA	NA
	Instances of bursae (0-9), erosion (0-5), synovial hypertrophy (0-5)	5 (3–8)	NA	NA
Morphological Measurements	Depth of tissue under MH1 (mm)	15.6 (12.9–18.1)	16.7	9
	Unloaded lateral offset of sesamoid from MH1 edge (% of MH1 width)	22.1 (17.3–32.9)	14.4	2 =
	Average principal curvature of MH1 (mm^−1^)	0.149 (0.128–0.163)	0.079	1
Model Predictions	99th% shear strain in limb (%)	13.7 (12.3–16.7)	12.3	5
	Volume of tissue over 10% shear strain (mm^3^)	3,214 (2,359–6,407)	1,418	4
	99th% plantar pressure (kPa)	62.9 (59.3–65.6)	55.6	4
	Maximum plantar pressure gradient (kPa/mm)	3.1 (2.6–3.4)	3.4	10

### FE Model Predictions Across Participants With RA

**FIGURE 5:**
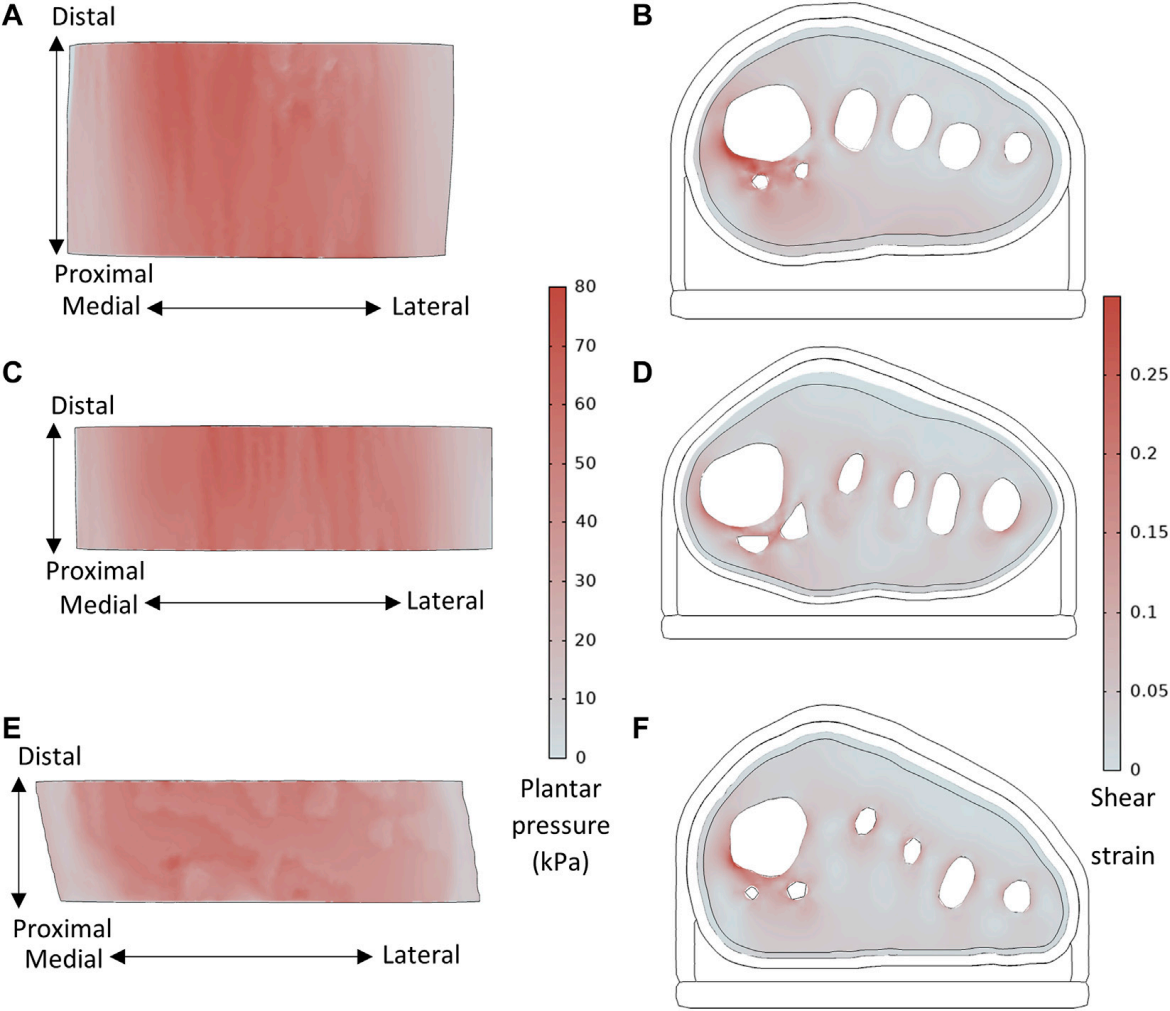
Plantar pressure and shear strain (taken from slice through sesamoid center) distributions, for **(A,B)** P8 with sesamoids in neutral position, BMI = 21.7, MH1 curvature = 0.144 mm^−1^, and **(C,D)** P1 with displaced sesamoids, BMI = 26.7, MH1 curvature = 0.112 mm^−1^, and **(E,F)** Healthy participant, BMI = 22.3, MH1 curvature = 0.079mm^−1^.

Across all participants, the highest plantar pressures were located under the first or second metatarsal heads (Figure 5). Peak shear strains were concentrated in the soft tissue around the bones, particularly the medial first metatarsal head aspect and sesamoid bones.

**FIGURE 6:**
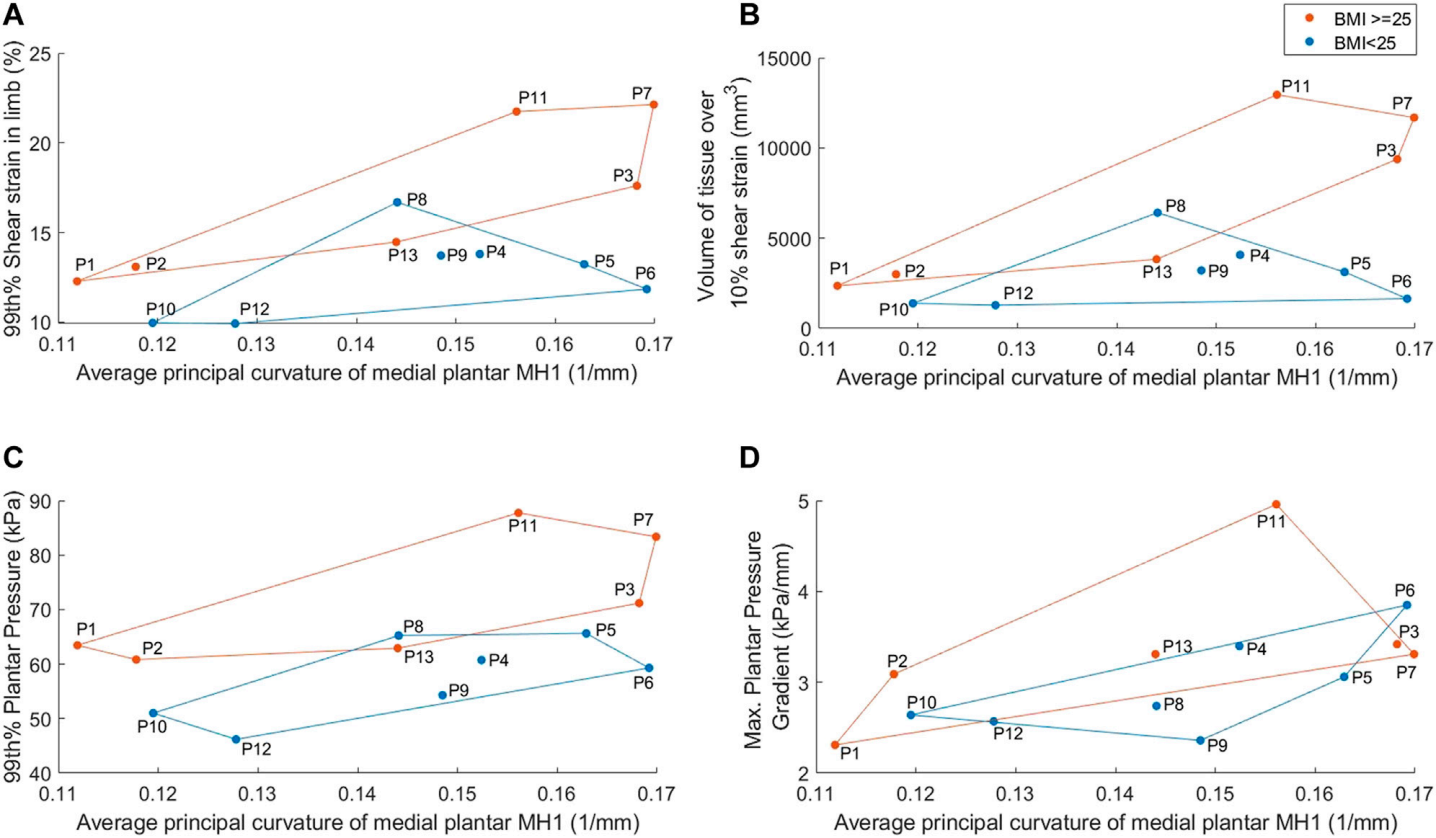
Average medial plantar MH1 curvature results, separated into normal vs high BMI groups, for: **(A)** 99th% shear strain in the limb **(B)** volume of tissue in the limb over 10% shear strain **(C)** 99th% plantar pressure **(D)** maximum plantar pressure gradient. Groups identified by marker color and convex hull boundaries.

The model results displayed differences between participants with RA for some parameters, with BMI and MH1 curvature appearing to have the greatest impact (Figure 6). The four participants with the highest BMIs all produced model predictions above the median values (P3, P7, P11, P13). There was no discernible trend in model results for the participants with the four lowest BMIs (P5, P6, P8, P9). Overall, there were strong significant correlations between BMI and 99th% shear strain, volume of tissue over 10% shear strain, and 99th% plantar pressure (0.600 < *r* < 0.652, *p* < 0.05) (Table 3).

**TABLE 3:** Correlations for model predictions with the clinical data and morphological measurements. Moderate to strong correlations (>0.4) are bolded. *indicates significant (*p* < 0.05) correlation.

Variable 1	Variable 2	Correlation (*p* value)
99th% Shear strain in limb	BMI	0.600 (0.030)*
	Unloaded lateral offset of sesamoid	-0.662 (0.014)*
	Tissue Depth under MH1	0.623 (0.023)*
	LFIS-IF	0.271 (0.370)
	Disease Duration	-0.601 (0.030)*
	Instances of bursae, erosion, synovial hypertrophy	0.284 (0.372)
	Average principal curvature of MH1	0.574 (0.040)*
Volume of tissue over 10% shear strain	BMI	0.652 (0.016)*
	Unloaded lateral offset of sesamoid	-0.758 (0.003)*
	Tissue Depth under MH1	0.627 (0.022)*
	LFIS-IF	0.222 (0.467)
	Disease Duration	-0.709 (0.007)*
	Instances of bursae, erosion, synovial hypertrophy	0.157 (0.627)
	Average principal curvature of MH1	**0.543 (0.055)**
99th% Plantar pressure	BMI	0.644 (0.018)*
	Unloaded lateral offset of sesamoid	-0.578 (0.038)*
	Tissue Depth under MH1	**0.464 (0.110)**
	LFIS-IF	0.323 (0.281)
	Disease Duration	-0.621 (0.023)*
	Instances of bursae, erosion, synovial hypertrophy	0.128 (0.691)
	Average principal curvature of MH1	**0.529 (0.063)**
Max. plantar pressure gradient	BMI	**0.452 (0.121)**
	Unloaded lateral offset of sesamoid	**-0.485 (0.093)**
	Tissue Depth under MH1	0.374 (0.208)
	LFIS-IF	0.126 (0.681)
	Disease Duration	-0.335 (0.264)
	Instances of bursae, erosion, synovial hypertrophy	-0.206 (0.520)
	Average principal curvature of MH1	0.557 (0.048)*

Similarly, three of the four participants with the lowest MH1 curvature (i.e., highest radius) accounted for three of the four lowest model predictions for strain and pressure gradient metrics (P1, P10, P12). Those with the four highest curvatures also had two to three of the highest pressure and strain predictions, with a clear distinction between those with high curvature and high BMI (P3, P7) and high curvature but normal BMI (P5, P6). Overall, MH1 curvature was moderately correlated with borderline significance to all four model predictions (0.529 < *r* < 0.574, 0.040 < *p* < 0.063). This was the only parameter to produce a significant correlation with the pressure gradients.

Sesamoid offset and tissue depth under MH1 also displayed moderate to strong correlations with the shear strain variables and to a lesser extent the 99th% plantar pressure (-0.578 < *r* < -0.758, *p* < 0.05 and 0.464 < *r* < 0.627, 0.022 < *p* < 0.110 respectively). This would suggest that higher pressure and strain results stemmed from reduced sesamoid offset and higher tissue depths, which would be associated with more normal anatomy. However, it should be noted that the four participants with the lowest sesamoid offsets (P2, P3, P11, P13) all had BMIs in the top five of the group, and those with the highest sesamoid offsets (P1, P6, P10, P12) all had either MH1 curvature or BMI within the lowest four of the group. Similar trends were found with the tissue depth variable.

Neither the LFIS-IF score or instances of bursae, erosion and synovial hypertrophy correlated with model predictions. Where there were potential trends for low or high rankings within the dataset, these could also be attributed to MH1 curvature and BMI. Full results for each participant, indicating the highest and lowest ranked participants for each variable, can be found in the Supplementary Data–Section A.

## Discussion

The aim of this study was to determine whether simplified computational forefoot models would produce differing biomechanical predictions depending on condition severity in people with RA, e.g. LFIS-IF score and morphological measures, or other relevant factors such as BMI. These predictions were also compared to model predictions from a healthy individual to determine any differences. Models capable of producing different predictions between these individuals would allow for personalised FO design to improve treatment. The model predictions differed between the foot MR data for a healthy individual and the data for those with RA, as seen in the pressure and strain results (Table 2). Comparing to a single healthy individual does not confirm the models would distinguish between cohorts of healthy vs RA, but the results do provide another point of comparison for condition severity. Within the RA group, higher BMIs corresponded to higher model predictions of soft tissue shear strain and plantar pressure, as did higher medial plantar MH1 curvature (indicating a less rounded, more sharply curved bone contour). The models could not distinguish between LFIS-IF score or instances of bursae, erosion and synovial hypertrophy. RA prevalence is significantly higher in women than men (Symmons et al., 2002), and all participants in this study were female. However, this does mean the findings are only applicable to women with RA and may not apply to men with RA. The predicted pressure and strain varied considerably across the participants, emphasizing the importance of evaluating multiple individuals. Previous studies using single cases or healthy cohorts are unlikely to provide a robust assessment of interventions.

The plantar pressures predicted by the models (median: 63kPa, IQR: 59–66kPa) fell within the expected bounds of forefoot pressures during midstance in the presence of an FO. Experimental testing of in-shoe midstance pressures of five healthy participants recorded median peak forefoot pressures of 131kPa (IQR: 95–151kPa), see Supplementary Data–Section B for details of the testing. Though higher than the pressure results in the present study, the experimental pressures were measured without an FO. FOs have been shown to reduce plantar pressures by 56% during stance (Kato et al., 1996), which would bring the experimental results far more in line with the model predictions. A recent study by Simonsen et al. (Simonsen et al., 2021) measured in-shoe plantar pressures for people with RA wearing orthoses, and found that at 50% of the stance phase, peak pressures ranged from approximately 17–54kPa, with a mean of around 29kPa with a custom FO. Previous studies of healthy individuals have found mean midstance forefoot pressures of approximately 70kPa (Aliberti et al., 2011) and 132kPa (s.d. 65kPa) (Kanatli et al., 2008), though these values were obtained barefoot which causes higher plantar pressures than when shod. Additionally, the shear strains in the present models were concentrated around the bones, with lower tissue strains elsewhere (Figure 5). These strain distributions are a well-established occurrence in the foot (Luboz et al., 2014), and correspond to common sites of ulceration due to RA (Firth et al., 2008). The models predicted results within the expected range, and produced trends based on participant clinical and morphological data. This is a promising sign that the models would be suitable for assessing FO and footwear choices across at least a female population, as the group represented in the present study.

One of the clearest trends observed was the effect of BMI on pressure and shear strain predictions, with high BMI (≥25 kg/m^2^) posing more risks. The differences in model results between participants were not just due to the applied loading conditions, which were based on participant weight. Three participants (P1, P3, P7) had identical weights and thus similar applied loads, but their BMIs differed as did the model results. Individuals with both RA and increased BMI experience increased pain, MTP joint swelling, activity limitation, and in-shoe pressures but little change to barefoot pressures (Dahmen et al., 2020). Thus, restricting the foot within a shoe caused more issues for those with higher BMIs, who may already be adversely affected due to higher loads going through the foot (Mickle and Steele, 2015). Additionally, people with high BMI may have different requirements for an FO to provide the necessary shock absorption. The importance of including the shoe in modelling of this nature is clear, and the present approach could be adapted for future FO design research in different groups at high risk of soft tissue injury in the foot, because the models can assess the effects of varying morphology, disease presentation and footwear choice.

The curvature of the medial plantar MH1 was also related to the model predictions. Individuals with a more rounded MH1 tended to produce lower model results and vice versa. The highest shear strains were also observed in the tissue surrounding this region. This was likely due to a combination of the medially skewed loading, and compression of the tissue between the bony prominence and orthosis/shoe. This parameter is not currently considered during FO or footwear assessments, but could provide additional information for such a use, particularly in identifying individuals with higher bone curvatures who may require more protection.

MH1 curvature was the only parameter that significantly correlated with the model pressure gradient predictions. Again, this likely relates to it being a bony prominence, where pressure gradients are higher and indicative of shear strain (Mueller et al., 2005; Lung et al., 2019). The lack of relationship between pressure gradients and other variables is understandable, particularly for BMI, given that the measure is not magnitude-based. Additionally, the FO may have reduced pressure gradients across all participants, including the healthy individual, limiting differences between them.

Increased tissue depth under MH1 and reduced sesamoid offset were also connected to increased pressure and shear strain predictions. However, this may have been an indirect effect due to BMI and MH1 curvatures. The majority of participants with longer disease durations had normal BMIs, while most with shorter durations had high BMIs (Figure 4B). Sesamoid offset increased with duration, so trends observed for normally positioned sesamoid bones may have been due to high BMIs instead, through artefacts of the small population. Similar overlaps were found with high sesamoid offset and low MH1 curvature. It should also be noted that the participants with highly displaced sesamoid bones (P1, P6, P10, P12) did not necessarily have worse conditions according to LFIS-IF scores and instances of bursae, erosion and synovial hypertrophy (Figure 4C, D).

The strength of this study was the consideration of these inhomogeneities and variations within the study population, however a few limitations should be acknowledged, which arise because the images used to develop the models were not originally collected for the purposes of simulation. First, the MR data used for the RA models had been collected in unloaded positions. Thus, the shape of the plantar foot varied considerably between participants, affecting the thickness of orthotic present in different forefoot regions, including where reduced tissue depth may have resulted in increased FO thickness (Figure 3). Given that FO thickness may influence pressure and strain (Goske et al., 2006; Chen et al., 2015), inter-participant comparisons may have been affected by differing FO thicknesses due to the varying plantar profiles. These limitations in the dataset make it difficult to draw conclusions on the effect of sesamoid offset and tissue depth under MH1 in these models. Further work with imaging collected in stance position but low, nominal loading, or a larger sample size, would be required to ascertain if these two RA-related variables were truly identifiable in participants’ model results. The small sample size in the present study may also have affected the other correlations, and so assessment of a larger cohort would confirm those results.

The models were not capable of distinguishing between participant’s LFIS-IF scores or instances of bursae, erosion or synovial hypertrophy, though there are possible explanations. First, the LFIS-IF score is a subjective measure, based on each individual’s perception of their experience and pain threshold. A clear example of this was P5, who’s LFIS-IF score was highest at 18 despite having no instances of bursae, erosion or synovial hypertrophy visible on ultrasound (Figure 4). Second, simplifications in the model geometries may not have allowed for distinguishing these parameters. Any bursae present were not included in the models, nor was detailed anatomy of the MTP joints. More complex models including these features may show relationships between the model results and the above parameters. The model simplifications, such as use of a bulk soft tissue group and fused MTP joints, were thought suitable for the purpose of this study and future purpose of the models which centers on comparisons between FO design, for which the absolute values of pressure and strain are not necessary. However, the simplifications may have limited the differences in predictions between the models.

Other model limitations stemmed from the loading conditions, such as the ground reaction forces that were applied to the models. As previously mentioned, a key improvement would be the use of forefoot MR data with the soft tissues and bones in a loaded position, as well as collecting kinetic data for the participants being modelled. In the present study, the GRFs applied to the models were based on a set load distribution across the forefoot, albeit scaled to the participant’s weight and relative locations of the metatarsal heads. Thus, the pressure and strain predictions may be inaccurate, particularly given that some individuals with RA adapt their gait to off-load painful or affected forefoot regions (Bowen et al., 2011; Carroll et al., 2015). To use these models to assess FO design on a personalised level, load distributions based on the individual’s gait pattern would be beneficial.

Additionally, using static midstance loads does not encompass the peak pressures experienced by the forefoot during toe-off, or the full sesamoid bone movement as would occur during dynamic gait influencing pressure and strain distributions. However, as conditions were consistent across participants, comparisons were still valid. Another drawback of using static models was that PTI differences between participants could not be examined. Given the importance of sustained loading and how it can relate to pain experienced by those with RA (van der Leeden et al., 2006), and risk of tissue damage (Gefen, 2009; Lung et al., 2016), dynamic modelling should also be explored.

## Conclusion

The model predictions for those with RA were highly influenced by the participant’s BMI and the medial plantar MH1 curvature. Due to limitations of the dataset, it was unclear whether the tissue depth under MH1 and the unloaded lateral offset of the sesamoids bone directly impacted model results. No relation was found between the model’s pressure or strain predictions and LFIS-IF score or instances of bursae, erosion and synovial hypertrophy. The wide ranges observed in the model predictions emphasizes the importance of modelling interventions across multiple pathological individuals rather than a single healthy case.

The simplified forefoot models produced differing biomechanical predictions between people with RA, with the variation relating to some condition-related factors but not to others. The models also produced differing results for a healthy individual and people with RA. Thus, with the limitations from the present study addressed, the models could provide a suitable basis for comparing FO designs based on individual requirements, particularly as they relate to BMI and alleviating internal tissue strains around bony prominences.

## Data Availability

The datasets presented in this study can be found in the University of Southampton repository: https://doi.org/10.5258/SOTON/D2061.

## Appendix 1: COMSOL Input Parameters Used for Meshing the Forefoot Models

This appendix details the mesh input parameters that were used to develop the FE models in the study (Table A1).

**TABLE A1:** Mesh input parameters.

Mesh input parameter	Region 1: Sock, skin	Region 2: Soft tissue and bone	Region 3: Orthosis upper boundary	Region 4: Orthosis and shoe sides	Region 5: Shoe sole
Maximum element size (mm)	1.68	4	1	5	5.5
Smallest element size allowed (mm)	1.68–1.68/3	4–4/3	1–1/3	5–5/3	5.5–5.5/3
Maximum element growth rate	1.3	1.3	1.3	1.3	1.3
Curvature factor	0.3	0.3	0.3	0.3	0.9
Resolution of narrow regions	1.2	1.2	1.2	1.2	0.4

## Appendix 2: Sensitivity Analysis

A sensitivity analysis was performed to ensure that the proximity of the forefoot section’s cut edges to the region of interest would not affect the model results. Example models were run, removing a coronal MR slice at a time from both edges of the data. It was determined that as long as there was a slice between the region of interest (e.g., the sesamoid bones) and the section edge, the results were unaffected by the edge proximity (<0.5% difference in percentile strain and pressure, Table A2).

**TABLE A2:** Results from the sensitivity analysis of removing coronal MR slices from the forefoot section model.

Model prediction parameter	Original model	Model with 1 slice removed from edges	Model with 2 slices removed from edges
99th% shear strain in limb (%)	12.28	12.33	11.88
Volume of limb over 10% shear strain (mm^3^)	2,359	2,392	1,406
99th% plantar pressure (kPa)	63.47	63.63	62.65
