# Supplementary material for: Predicting Forefoot-Orthosis Interactions in Rheumatoid Arthritis Using Computational Modelling
Source: Front Bioeng Biotechnol. 2021 Dec 23;9:803725. doi: 10.3389/fbioe.2021.803725 (PMC8733946; doi:10.3389/fbioe.2021.803725)
Supplement: Supplementary file 1 [file DataSheet1.DOCX]

Supplementary Material

# Section A

This document contains the full results for the:

- Clinical data for each participant (Table 1)
- Morphological measurements for each participant (Table 2)
- Computational model predictions for each participant (Table 3)
- Relevant correlations between clinical data and morphological measurements (Table 4)

Table 1: Clinical data for each participant

| **Participant** | **BMI** | **Disease Duration (years)** | **LFIS-IF Score** | **Instances of bursae between and below joints** | **Instances of erosion at joints** | **Instances of synovial hypertrophy at joints** | **Total instances of bursae, erosion, hypertrophy** |
| --- | --- | --- | --- | --- | --- | --- | --- |
| **P1** | 26.7 | 14 | 13 | 4 | 3 | 1 | 8 |
| **P2** | 28.3 | 8 | 12 | 4 | 0 | 0 | 4 |
| **P3** | 30.9 | 1 | 5 | 1 | 1 | 1 | 3 |
| **P4** | 23.2 | 2 | 9 | 1 | 0 | 5 | 6 |
| **P5** | 21.7 | 3 | 18 | 0 | 0 | 0 | 0 |
| **P6** | 22.3 | 30 | 5 | 0 | 1 | 2 | 3 |
| **P7** | 29.5 | 4 | 13 | 2 | 1 | 5 | 8 |
| **P8** | 21.7 | 22 | 15 | 2 | 4 | 2 | 8 |
| **P9** | 22.1 | 12 | 7 | 1 | 4 | 2 | 7 |
| **P10** | 24.8 | 35 | 4 | 1 | 0 | 0 | 1 |
| **P11** | 30.2 | 5 | 17 | 0 | 3 | 1 | 4 |
| **P12** | 22.9 | 28 | 16 | NA | NA | NA | NA |
| **P13** | 28.4 | 6 | 15 | 4 | 4 | 2 | 10 |

Table 2: Morphological measurements per participant, taken from MR data. For participants with RA: green indicates ranking 1-4 (lowest scores) and red indicates ranking 10-13 (highest scores)

| **Participant** | **Sesamoid Position (Normal/Displaced)** | **Unloaded lateral offset of sesamoid from MH1 edge (as % of MH1 width)** | **Depth of Tissue under MH1 (mm)** | **Average Principal Curvature of MH1 (mm^-1^)** |
| --- | --- | --- | --- | --- |
| **P1** | Displaced | 40.0 | 12.9 | 0.112 |
| **P2** | Normal | 17.3 | 10.9 | 0.118 |
| **P3** | Normal | 14.2 | 18.8 | 0.168 |
| **P4** | Normal | 17.8 | 16.2 | 0.152 |
| **P5** | Normal | 29.0 | 14.1 | 0.163 |
| **P6** | Displaced | 39.8 | 12.5 | 0.169 |
| **P7** | Normal | 20.2 | 15.1 | 0.170 |
| **P8** | Normal | 23.4 | 19.8 | 0.144 |
| **P9** | Normal | 22.1 | 17.6 | 0.149 |
| **P10** | Displaced | 72.0 | 9.9 | 0.120 |
| **P11** | Normal | 5.7 | 20.8 | 0.156 |
| **P12** | Displaced | 32.9 | 15.6 | 0.128 |
| **P13** | Normal | 14.0 | 18.1 | 0.144 |
| **Healthy** | Normal | 14.4 | 16.7 | 0.0786 |

Table 3: Results from computational models per participant. For participants with RA: green indicates ranking 1-4 (lowest scores) and red indicates ranking 10-13 (highest scores)

| **Participant** | **99^th^% Shear strain in limb (%)** | **Volume of tissue over 10% shear strain (mm^3^)** | **99^th^% Plantar pressure (kPa)** | **Max. plantar pressure gradient (kPa/mm)** |
| --- | --- | --- | --- | --- |
| **P1** | 12.28 | 2359 | 63.47 | 2.31 |
| **P2** | 13.09 | 2999 | 60.84 | 3.09 |
| **P3** | 17.60 | 9377 | 71.18 | 3.42 |
| **P4** | 13.80 | 4078 | 60.76 | 3.40 |
| **P5** | 13.23 | 3124 | 65.62 | 3.06 |
| **P6** | 11.84 | 1641 | 59.31 | 3.85 |
| **P7** | 22.13 | 11686 | 83.37 | 3.31 |
| **P8** | 16.68 | 6407 | 65.24 | 2.74 |
| **P9** | 13.72 | 3214 | 54.30 | 2.36 |
| **P10** | 9.96 | 1386 | 51.01 | 2.64 |
| **P11** | 21.74 | 12955 | 87.76 | 4.96 |
| **P12** | 9.92 | 1285 | 46.19 | 2.57 |
| **P13** | 14.47 | 3833 | 62.94 | 3.31 |
| **Healthy** | 12.33 | 1418 | 55.57 | 3.37 |

Table 4: Correlations for clinical data and morphological measurements. Moderate to strong correlations (>0.4) are bolded. * indicates significant (*p*<0.05) correlation.

| **Variable 1** | **Variable 2** | **Correlation (*p* value)** |
| --- | --- | --- |
| Disease duration | Unloaded lateral offset of sesamoid | **0.698 (0.008)*** |
|  | Tissue Depth under MH1 | **-0.428 (0.145)** |
|  | LFIS-IF | -0.309 (0.304) |
|  | Instances of bursae, erosion, synovial hypertrophy | -0.198 (0.537) |
|  | Average principal curvature of MH1 | **-0.401 (0.174)** |
| LFIS-IF Score | Unloaded lateral offset of sesamoid | **-0.425 (0.148)** |
|  | Tissue Depth under MH1 | 0.332 (0.267) |
|  | Instances of bursae, erosion, synovial hypertrophy | 0.259 (0.417) |
|  | Average principal curvature of MH1 | -0.069 (0.823) |
| Instances of bursae, erosion, synovial hypertrophy | Tissue Depth under MH1 | **0.406 (0.190)** |
|  | Average principal curvature of MH1 | -0.124 (0.701) |
| Tissue Depth under MH1 | Unloaded lateral offset of sesamoid | **-0.721 (0.005)*** |

# Section B

## Methods

Five healthy participants (2 male, 3 female) were recruited to examine plantar pressures during gait. Ethical approval was granted by the University of Southampton (ERGO ID: 51969). Each participant walked at a self-selected pace on a treadmill for 2 minutes, whilst wearing training shoes of an appropriate size that were provided for them (NDB Lightweight Cross-training running trainers). In-shoe plantar pressures were sampled at 50Hz using F-scan sensors (Tekscan, Massachusetts, USA) throughout the testing.

The F-scan Research Software (v.7.5, Tekscan, USA) was then used to extract the peak pressures experienced by both feet in different foot regions during midstance. The 10th stance was used for the analysis for each participant – this was chosen arbitrarily from the middle of the test condition. Midstance was identified and the peak pressures in the forefoot from that time point were recorded. These peak pressures were then used for comparison to the model results to determine whether the midstance pressures predicted by the models were within the expected range.

## Results

Table 5: Peak forefoot pressures measured during midstance of gait

|  | **Peak pressures in Forefoot (kPa)** | |
| --- | --- | --- |
| **Participant** | **Left foot** | **Right foot** |
| P1 | 105 | 85 |
| P2 | 352 | 176 |
| P3 | 133 | 92 |
| P4 | 151 | 129 |
| P5 | 151 | 70 |
